# Utilizing large language models in breast cancer management: systematic review

**DOI:** 10.1007/s00432-024-05678-6

**Published:** 2024-03-19

**Authors:** Vera Sorin, Benjamin S. Glicksberg, Yaara Artsi, Yiftach Barash, Eli Konen, Girish N. Nadkarni, Eyal Klang

**Affiliations:** 1 Department of Diagnostic Imaging, Chaim Sheba Medical Center, Affiliated to the Sackler School of Medicine, Tel-Aviv University, Emek Haela St. 1, 52621 Ramat Gan, Israel; 2 DeepVision Lab, Chaim Sheba Medical Center, Tel Hashomer, Israel; 3 Division of Data-Driven and Digital Medicine (D3M), Icahn School of Medicine at Mount Sinai, New York, NY USA; 4 Azrieli Faculty of Medicine, Bar-Ilan University, Zefat, Israel; 5 The Charles Bronfman Institute of Personalized Medicine, Icahn School of Medicine at Mount Sinai, New York, NY USA

**Keywords:** Large language models, GPT, Breast cancer, Artificial intelligence

## Abstract

**Purpose:**

Despite advanced technologies in breast cancer management, challenges remain in efficiently interpreting vast clinical data for patient-specific insights. We reviewed the literature on how large language models (LLMs) such as ChatGPT might offer solutions in this field.

**Methods:**

We searched MEDLINE for relevant studies published before December 22, 2023. Keywords included: “large language models”, “LLM”, “GPT”, “ChatGPT”, “OpenAI”, and “breast”. The risk bias was evaluated using the QUADAS-2 tool.

**Results:**

Six studies evaluating either ChatGPT-3.5 or GPT-4, met our inclusion criteria. They explored clinical notes analysis, guideline-based question-answering, and patient management recommendations. Accuracy varied between studies, ranging from 50 to 98%. Higher accuracy was seen in structured tasks like information retrieval. Half of the studies used real patient data, adding practical clinical value. Challenges included inconsistent accuracy, dependency on the way questions are posed (prompt-dependency), and in some cases, missing critical clinical information.

**Conclusion:**

LLMs hold potential in breast cancer care, especially in textual information extraction and guideline-driven clinical question-answering. Yet, their inconsistent accuracy underscores the need for careful validation of these models, and the importance of ongoing supervision.

**Supplementary Information:**

The online version contains supplementary material available at 10.1007/s00432-024-05678-6.

## Introduction

Natural language processing (NLP) is increasingly used in healthcare, especially in oncology, for its ability to analyze free-text with diverse applications (Sorin et al. 2020a, b). Large language models (LLMs) like GPT, LLaMA, PaLM, and Falcon represent the pinnacle of this development, leveraging billions of parameters for highly accurate text processing (Sorin et al. 2020a, b; Bubeck et al. 2023). Despite this, the integration of such sophisticated NLP algorithms into practical healthcare settings, particularly in managing complex diseases like breast cancer, remains a technological, operational, and ethical challenge.

Breast cancer, the most common cancer among women, continues to pose significant challenges in terms of morbidity, mortality, and information overload (Kuhl et al. 2010; Siegel et al. 2019). While LLMs have shown promise in medical text analysis—with GPT-4 achieving a notable 87% success rate on the USMLE (Brin et al. 2023; Chaudhry et al. 2017) and extending its capabilities to image analysis (Sorin et al. 2023a, b, c)—their practical application in medicine and oncology in particular is still evolving.

We reviewed the literature on how large language models (LLMs) might offer solutions in breast cancer care.

## Methods

This review was conducted according to the Preferred Reporting Items for Systematic Reviews and Meta-Analyses (PRISMA) guidelines (Moher 2009). We searched the literature for applications of LLMs in breast cancer management using MEDLINE.

The search included studies published up to December 22nd 2023. Our search query was “(("large language models") OR (llm) OR (gpt) OR (chatgpt) OR (openAI)) AND (breast)”. The initial search identified 97 studies. To ensure thoroughness, we also examined the reference lists of the relevant studies. This, however, did not lead to additional relevant studies that met our inclusion criteria.

The criteria for inclusion were English language full-length publications that specifically evaluated the role of LLMs in breast cancer management. We excluded papers that addressed other general applications of LLMs in healthcare or oncology without a specific focus on breast cancer.

**Fig. 1:**
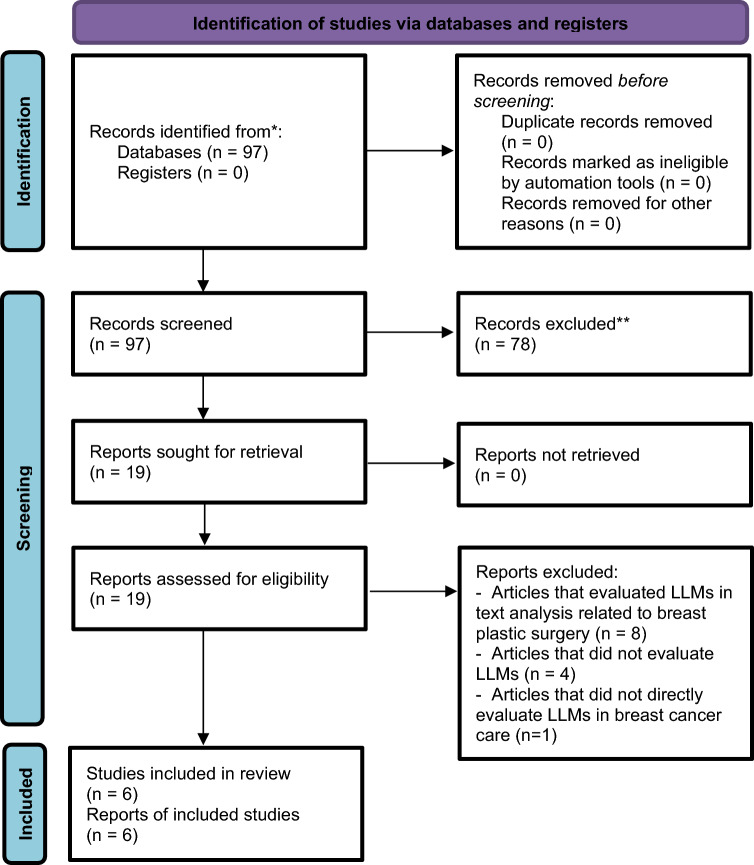
Flow Diagram of the Inclusion Process. Flow diagram of the search and inclusion process based on the Preferred Reporting Items for Systematic Reviews and Meta-Analyses (PRISMA) guidelines

Three reviewers (VS, YA, and EKL) independently conducted the search, screened the titles, and reviewed the abstracts of the articles identified in the search. One discrepancy in the search results was discussed and resolved to achieve a consensus. Next, the reviewers assessed the full text of the relevant papers. In total, six publications met our inclusion criteria and were incorporated into this review. We summarized the results of the included studies, detailing the specific LLMs used, the utilized tasks, number of cases, along with publication details in a table format. Figure 1 provides a flowchart detailing the screening and inclusion procedure.

Quality was assessed by the Quality Assessment of Diagnostic Accuracy Studies (QUADAS-2) criteria (Whiting 2011).

## Results

All six studies included were published in 2023 (Table 1). All focused on either ChatGPT-3.5 or GPT-4 by OpenAI. Applications described include information extraction and question-answering. Three studies (50.0%) evaluated the performance of ChatGPT on actual patient data (Sorin et al. 2023a, b, c; Choi et al. 2023; Lukac et al. 2023), as opposed to two studies that used data from the internet (Rao et al. 2023; Haver et al. 2023). One study crafted fictitious patient profiles by the head investigator (Griewing et al. 2023).

**Table 1:** Studies evaluating LLMs for breast cancer diagnosis and care

Study ^ref^	Publication Date	Title	Journal
Sorin et al. 2023	05.2023	Large language model (ChatGPT) as a support tool for breast tumor board	NPJ Breast Cancer
Rao et al. 2023	06.2023	Evaluating GPT as an Adjunct for Radiologic Decision Making: GPT-4 Versus GPT-3.5 in a Breast Imaging Pilot	JACR
Choi et al. 2023	09.2023	Developing prompts from large language model for extracting clinical information from pathology and ultrasound reports in breast cancer	Radiation Oncology Journal
Lukac et al. 2023	07.2023	Evaluating ChatGPT as an adjunct for the multidisciplinary tumor board decision-making in primary breast cancer cases	Archives of Gynecology and Obstetrics
Haver et al. 2023	04.2023	Appropriateness of Breast Cancer Prevention and Screening Recommendations Provided by ChatGPT	Radiology
Griewing et al. 2023	10.2023	Challenging ChatGPT 3.5 in Senology—An Assessment of Concordance with Breast Cancer Tumor Board Decision Making	Journal of Personalized Medicine

**Fig. 2:**
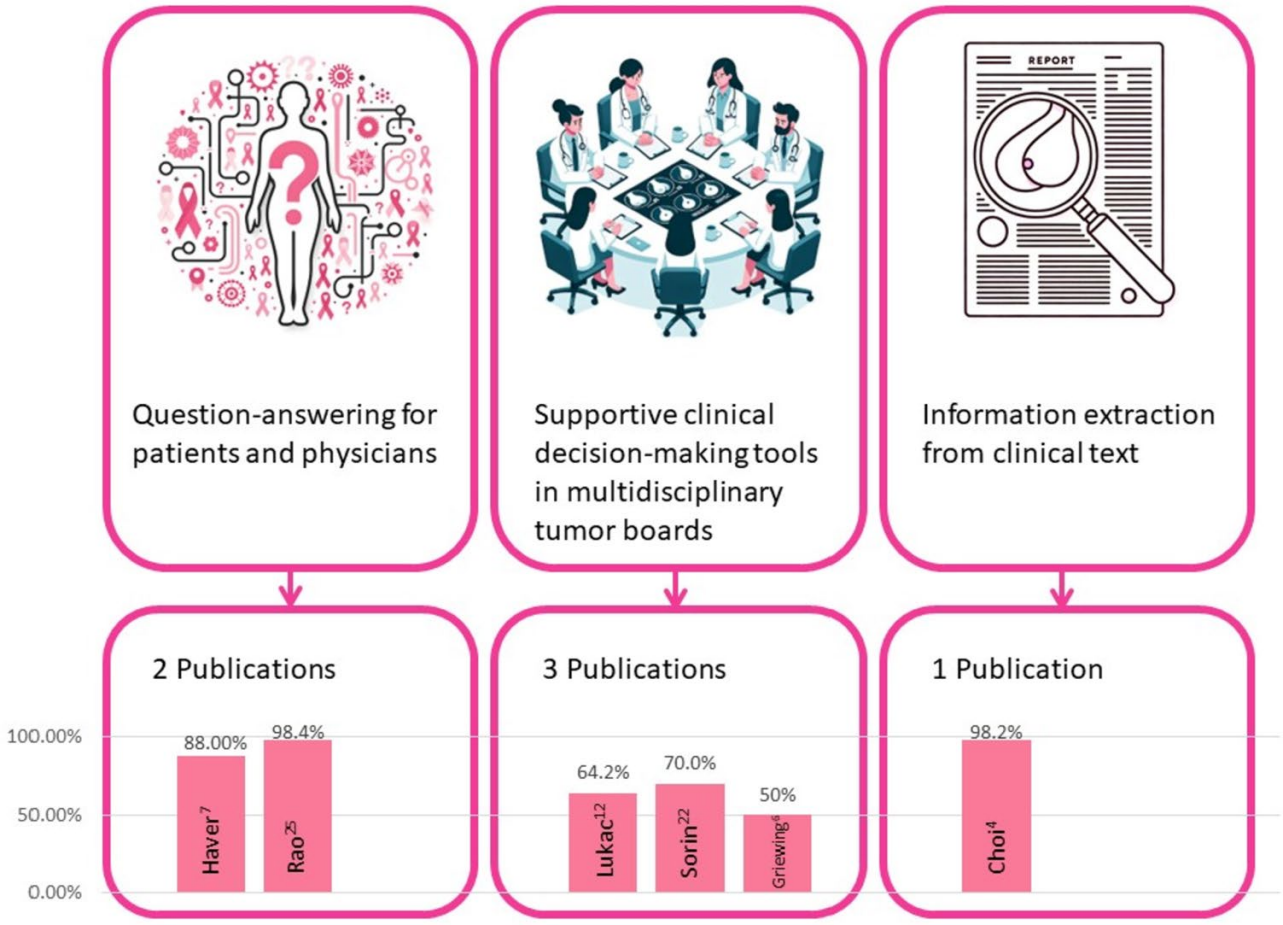
Applications of large language models in breast cancer care and the corresponding accuracies achieved in various tasks in the different studies

Rao et al. and Haver et al. evaluated LLMs for breast imaging recommendations (Rao et al. 2023; Haver et al. 2023). Sorin et al., Lukac et al. and Griewing et al. evaluated LLMs as supportive decision-making tools in multidisciplinary tumor boards (Sorin et al. 2023a, b, c; Lukac et al. 2023; Griewing et al. 2023). Choi et al. used LLM for information extraction from ultrasound and pathology reports (Choi et al. 2023) (Fig. 2). Example cases for applications from studies are detailed in Table 2.

**Table 2:** Example cases

Study ^ref^	Application	Example
Sorin et al. 2023	Breast tumor board clinical decision support	User: Hi, can I give you a patient story of breast cancer detected and you'll say what is the next step in her management? please decide if she needs surgery, what type of surgery, or whether she needs neo-adjuvant therapy before, or does she need further testing ChatGPT-3.5: Of course! I'd be happy to help. Please go ahead and give me the patient's story User: Here is the story: 51 years old woman, no family hx, no hormonal meds. felt a mass in her left breast and did mammography and US. On US of her left breast in the outer-upper quadrant a 2 cm mass was detected. Then, a biopsy was performed to the mass and on pathology it was an IDC ER positive, PR negative, HER2 negative, KI high. Then she did an MRI for local staging—a solitary mass in her left breast was detected. She also did PET-CT with no evidence of distal metastases ChatGPT-3.5: Based on the information provided, it appears that this patient has been diagnosed with Invasive Ductal Carcinoma (IDC) in her left breast Given the results of the pathology and imaging tests, the patient is most likely a candidate for surgery, as the mass is located in only one breast and there is no evidence of distant metastases For this type of breast cancer, the standard surgical options are either a lumpectomy (also known as a breast-conserving surgery) or a mastectomy (removal of the entire breast). In general, a lumpectomy is recommended for small, early-stage tumors, while a mastectomy may be recommended for larger tumors or for patients who have a higher risk of cancer recurrence In this case, given the size of the mass (2 cm), a lumpectomy with axillary lymph node dissection (removal of some of the lymph nodes in the underarm area to check for spread of cancer) would be the typical surgical approach. If a lumpectomy is performed, radiation therapy would usually be recommended to reduce the risk of recurrence It's worth noting that the patient's ER (estrogen receptor) positive, PR (progesterone receptor) negative, and HER2 (human epidermal growth factor receptor 2) negative status will play a role in determining her treatment options, as these receptors can be targeted with specific medications In conclusion, given the patient's diagnosis and test results, surgery, most likely a lumpectomy with axillary lymph node dissection, is the next recommended step in her management. After the surgery, the patient may also require additional treatment such as radiation therapy or systemic therapy (such as hormone therapy or chemotherapy) based on the results of the surgery and further testing
Rao et al. 2023	Question-answering based on ACR recommendations	Prompt: For variant "Breast cancer screening. Average-risk women: women with < 15% lifetime risk of breast cancer.", assess appropriateness of the following procedures in a concise manner: Mammography screening, Digital breast tomosynthesis screening, US breast, MRI breast without and with IV contrast, MRI breast without IV contrast, FDG-PET breast dedicated, Sestamibi MBI Mammography screening and Digital Breast Tomosynthesis screening are appropriate for average-risk women as these are the current standard screening methods for breast cancer ChatGPT: Ultrasound breast screening is also appropriate for average-risk women, as it is often used as an adjunctive test to mammography in women with dense breast tissue Magnetic Resonance Imaging (MRI) of the breast without and with IV contrast is generally not recommended for average-risk women as a screening tool. It may be considered for women with a high lifetime risk of breast cancer (usually defined as 20% or greater) Fluorodeoxyglucose (FDG) Positron Emission Tomography (PET) breast dedicated, and Sestamibi breast (MBI) is not standardly recommended as a screening modality for average-risk women, usually reserved for women with high-risk or recurrent breast cancer

Accuracy of LLMs on different applications ranged from 50 to 98%. Best performance rates were achieved for information extraction and question-answering, with correct responses ranging from 88 to 98% (Choi et al. 2023; Rao et al. 2023) (Table 3). The lower performance was for clinical decision support in breast tumor boards, ranging between 50 and 70% (Sorin et al. 2023a, b, c; Lukac et al. 2023; Griewing et al. 2023). The range in performance on this task was wide between studies. However, the methods of the three studies also varied significantly (Sorin et al. 2023a, b, c; Lukac et al. 2023; Griewing et al. 2023). Sorin et al. and Lukac et al. used authentic patient data and compared ChatGPT-3.5 to the retrospective decisions in breast tumor board (Sorin et al. 2023a, b, c; Lukac et al. 2023). In both studies, the authors used reviewers that scored ChatGPT-3.5 responses (Sorin et al. 2023a, b, c; Lukac et al. 2023). (Griewing et al. 2023) crafted 20 fictitious patient files that were then discussed by a multidisciplinary tumor board. Their assessment was based on binary evaluation of various treatment approaches, including surgery, endocrine, chemotherapy, and radiation therapy. Griewing et al. were the only study providing insights into LLM performance on genetic testing for breast cancer treatment (Griewing et al. 2023). All three studies analyzed concordance between the tumor board and the LLM on different treatment options (Sorin et al. 2023a, b, c; Lukac et al. 2023; Griewing et al. 2023).

**Table 3:** Summarization of performance of LLMs at different breast cancer care related tasks

Study ^ref^	LLM	No. of cases	Actual patient data	Application	Correct performance
Sorin et al. 2023	ChatGPT (GPT-3.5)	10	Yes	Tumor board clinical decision support	70%
Rao et al. 2023	GPT-4, GPT-3.5	14	No	Question-answering based on ACR recommendations	88.9–98.4%
Choi et al. 2023	ChatGPT (GPT-3.5)	340	Yes	Information extraction	87.7–98.2%
Lukac et al. 2023	ChatGPT (GPT-3.5)	10	Yes	Tumor board clinical decision support	64.20%
Haver et al. 2023	ChatGPT (GPT-3.5)	25	No	Question-answering on breast cancer prevention and screening	88%
Griewing et al. 2023	ChatGPT (GPT-3.5)	20	No	Concordance to tumor board clinical decisions	50–95%

All studies discussed the limitations of LLMs in the contexts in which the algorithms were evaluated (Table 4). In all studies some of the information the models generated was false. When used as a support tool for tumor board, in some instances, the models overlooked relevant clinical details (Sorin et al. 2023a, b, c; Lukac et al. 2023; Griewing et al. 2023). Sorin et al. noticed absolute lack of referral to imaging (Sorin et al. 2023a, b, c), while Rao et al. who evaluated appropriateness of imaging noticed imaging overutilization (Rao et al. 2023). Some of the studies also discussed whether the nature of the prompt affects the outputs (Choi et al. 2023; Haver et al. 2023), and the difficulty to verify the reliability of the answers (Lukac et al. 2023; Rao et al. 2023; Haver et al. 2023).

**Table 4:** Limitations of LLMs as described in each study

Study ^ref^	LLM	Limitations described
Sorin et al. 2023	ChatGPT (GPT-3.5)	False answers and inaccurate medical recommendations, overlooked relevant clinical details, absolute lack of referral to imaging, potential for outdated information, potential for bias
Rao et al. 2023	GPT-4, GPT-3.5	False information, imaging overutilization, lack of source attribution
Choi et al. 2023	ChatGPT (GPT-3.5)	False information, lack of logical reasoning, incomplete information extraction, prompt sensitivity
Lukac et al. 2023	ChatGPT (GPT-3.5)	False answers, overlooked relevant clinical details, potential for outdated information, lack of source attribution
Haver et al. 2023	ChatGPT (GPT-3.5)	False recommendations, prompt sensitivity, lack of source attribution
Griewing et al. 2023	ChatGPT (GPT-3.5)	Lack of Consistency in Health Data Use, treatment mistakes, prone to misinterpretation and hallucinations

According to the QUADAS-2 tool, all papers but one scored as high risk of bias for index test interpretation. For the paper by Lukac et al. the risk was unclear, refraining from a clear statement whether the evaluators were blinded to the reference standard. The study by Griewing et al. was the only one identified to have a low risk of bias across all categories (Griewing et al. 2023). The objective assessment of the risk of bias is reported in Supplementary Table 1.

## Discussion

We reviewed the literature on LLMs applications related to breast cancer management and care. Applications described included information extraction from clinical texts, question-answering for patients and physicians, manuscript drafting and clinical management recommendations.

A disparity in performance was seen. The models showed proficiency in information extraction and responding to structured questions, with accuracy rates between 88 and 98%. However, their effectiveness diminished down to 50–70% in making clinical decisions, underscoring a gap in their application. In breast cancer care, attention to detail is crucial. LLMs excel at processing medical information quickly. However, currently, they may be less adept at navigating complex treatment decisions. Breast cancer cases vary greatly, each case distinguished by a unique molecular profile, clinical staging, and patient-specific requirements. It is vital for LLMs to adapt to the individual patient. While these models can assist physicians in routine tasks, they require further development for personalized treatment planning.

Interestingly, half of the studies included real patients’ data as opposed to publicly available data or fictitious data. For the overall published literature on LLMs in healthcare, there are more publications evaluating performance on public data. This includes performance on board examinations and question-answering based on guidelines (Sallam 2023). These analyses may introduce contamination of data, since LLMs were trained on vast data from the internet. For commercial models such as ChatGPT, the type of training data is not disclosed. Furthermore, these applications do not necessarily reflect on the performance of these models in real-world clinical settings.

While some claim that LLMs may eventually replace healthcare personnel, currently, there are major limitations and ethical concerns that strongly suggest otherwise (Lee et al. 2023). Using such models to augment physicians’ performance is more practical, albeit also constrained by ethical issues (Shah et al. 2023). LLMs enable automating different tasks that traditionally required human effort. The ability to analyze, extract and generate meaningful textual information could potentially decrease some physicians’ workload and human errors.

Reliance on LLMs and potential integration in medicine should be made with caution. The limitations discussed in the studies further underscore this note. These models can generate false information (termed “hallucination”) which can be seamlessly and confidently integrated into real information (Sorin et al. 2020a, b). They can also perpetuate disparities in healthcare (Sorin et al. 2021; Kotek et al. 2023). The inherent inability to trace the exact decision-making process of these algorithms is a major challenge for trust and clinical integration (Sorin et al. 2023a, b, c). LLMs can also be vulnerable to cyber-attacks (Sorin et al. 2023a, b, c).

Furthermore, this study highlights the absence of uniform assessment methods for LLMs in healthcare, underlining the need of establishing methodological standards for evaluating LLMs. The goal is to enhance the comparability and quality of research. The establishment of such standards is critical for the safe and effective integration of LLMs into healthcare, especially for complex conditions like breast cancer, where personalized patient care is essential.

This review has several limitations. First, due to the heterogeneity of tasks evaluated in the studies, we could not perform a meta-analysis. Second, all included studies assessed ChatGPT-3.5, and only one study evaluated GPT-4. There were no publications identified on other available LLMs. Finally, generative AI is currently a rapidly expanding topic. Thus, there may be manuscripts and applications published after our review was performed. LLMs are continually being refined, and so is their performance.

To conclude, LLMs hold potential for breast cancer management, especially in text analysis and guideline-driven question-answering. Yet, their inconsistent accuracy warrants cautious use, following thorough validation and ongoing supervision.

## Supplementary Information

Below is the link to the electronic supplementary material.

Supplementary file1 (DOCX 19 KB)

## Data Availability

Reviewed studies and their results can be located at PubMed database: https://pubmed.ncbi.nlm.nih.gov/
