# Supplementary material for: Utilizing large language models in breast cancer management: systematic review
Source: J Cancer Res Clin Oncol. 2024 Mar 19;150(3):140. doi: 10.1007/s00432-024-05678-6 (PMC10950983; doi:10.1007/s00432-024-05678-6)
Supplement: Supplementary file 1 — Supplementary file1 (DOCX 19 KB) [file 432_2024_5678_MOESM1_ESM.docx]

**Supplementary Online Content**

**Supplementary Table 1.** Quality Assessment of Diagnostic Accuracy Studies.......2

**References**………………………………………………………………..…..……..3

This supplementary material has been provided by the authors to give readers additional information about the work.

**Supplementary Table 1: Quality Assessment of Diagnostic Accuracy Studies-2 (QUADS-2)**

Abbreviations: Pt. patient; Ref. reference. ✘ = high risk of bias; ✔ = low risk of bias. **?** = unclear risk. N/A – not applicable,

|  | RISK OF BIAS | | | | APPLICABILITY CONCERNS | | |
| --- | --- | --- | --- | --- | --- | --- | --- |
| **First Author** | **Patient Selection** | **Index  Test** | **Reference Standard** | **Flow and Timing** | **Patient Selection** | **Index  Test** | **Reference Standard** |
| Sorin et al.^1^ | ✔ | ✘ | ✔ | ✔ | ✔ | ✔ | ✔ |
| Rao et al.^2^ | N/A | ✘ | ✔ | ✔ | ✔ | ✔ | ✔ |
| Choi et al.^3^ | ✔ | ✘ | ✔ | ✔ | ✔ | ✔ | ✔ |
| Lukac et al.^4^ | ✔ | **?** | ✔ | ✔ | ✔ | ✔ | ✔ |
| Haver et al.^5^ | N/A | ✘ | ✘ | ✔ | ✔ | ✔ | ✘ |
| Griewing et al. | ✔ | ✔ | ✔ | ✔ | ✔ | ✔ | ✔ |

**References:**

1. Sorin V, Klang E, Sklair-Levy M, et al. Large language model (ChatGPT) as a support tool for breast tumor board. *npj Breast Cancer.* 2023;9(1).
2. Rao A, Kim J, Kamineni M, et al. Evaluating GPT as an Adjunct for Radiologic Decision Making: GPT-4 Versus GPT-3.5 in a Breast Imaging Pilot. *Journal of the American College of Radiology.* 2023.
3. Choi HS, Song JY, Shin KH, Chang JH, Jang B-S. Developing prompts from large language model for extracting clinical information from pathology and ultrasound reports in breast cancer. *Radiation Oncology Journal.* 2023;41(3):209-216.
4. Lukac S, Dayan D, Fink V, et al. Evaluating ChatGPT as an adjunct for the multidisciplinary tumor board decision-making in primary breast cancer cases. *Archives of Gynecology and Obstetrics.* 2023;308(6):1831-1844.
5. Haver HL, Ambinder EB, Bahl M, Oluyemi ET, Jeudy J, Yi PH. Appropriateness of Breast Cancer Prevention and Screening Recommendations Provided by ChatGPT. *Radiology.* 2023;307(4).
6. Griewing S, Gremke N, Wagner U, Lingenfelder M, Kuhn S, Boekhoff J. Challenging ChatGPT 3.5 in Senology-An Assessment of Concordance with Breast Cancer Tumor Board Decision Making. J Pers Med. 2023 Oct 16;13(10):1502. doi: 10.3390/jpm13101502. PMID: 37888113; PMCID: PMC10608120.
